# Neutrophil extracellular traps formation and clearance is enhanced in fever and attenuated in hypothermia

**DOI:** 10.3389/fimmu.2023.1257422

**Published:** 2023-10-02

**Authors:** Jakub Janko, Emil Bečka, Katarína Kmeťová, Letícia Hudecová, Barbora Konečná, Peter Celec, Mona Bajaj-Elliott, Michal Pastorek

**Affiliations:** ^1^ Institute of Molecular Biomedicine, Faculty of Medicine, Comenius University, Bratislava, Slovakia; ^2^ Institute of Pathophysiology, Faculty of Medicine, Comenius University, Bratislava, Slovakia; ^3^ Great Ormond Street Institute of Child Health, University College London, London, United Kingdom

**Keywords:** fever, hypothermia, neutrophil extracellular trap, polymorphonuclear cell, bacteria, mitochondria, DNase I

## Abstract

Fever and hypothermia represent two opposite strategies for fighting systemic inflammation. Fever results in immune activation; hypothermia is associated with energy conservation. Systemic Inflammatory Response Syndrome (SIRS) remains a significant cause of mortality worldwide. SIRS can lead to a broad spectrum of clinical symptoms but importantly, patients can develop fever or hypothermia. During infection, polymorphonuclear cells (PMNs) such as neutrophils prevent pathogen dissemination through the formation of neutrophil extracellular traps (NETs) that ensnare and kill bacteria. However, when dysregulated, NETs also promote host tissue damage. Herein, we tested the hypothesis that temperature modulates NETs homeostasis in response to infection and inflammation. NETs formation was studied in response to infectious (*Escherichia coli*, *Staphylococcus aureus*) and sterile (mitochondria) agents. When compared to body temperature (37°C), NETs formation increased at 40°C; interestingly, the response was stunted at 35°C and 42°C. While CD16+ CD49d+ PMNs represent a small proportion of the neutrophil population, they formed ~45-85% of NETs irrespective of temperature. Temperature increased formyl peptide receptor 1 (FPR1) expression to a differential extent in CD16+ CD49d- *vs*. CD49d+ PMNSs, suggesting further complexity to neutrophil function in hypo/hyperthermic conditions. The capacity of NETs to induce Toll-like receptor 9 (TLR9)-mediated NF-κB activation was found to be temperature independent. Interestingly, NET degradation was enhanced at higher temperatures, which corresponded with greater plasma DNase activity in response to temperature increase. Collectively, our observations indicate that NETs formation and clearance are enhanced at 40°C whilst temperatures of 35°C and 42°C attenuate this response. Targeting PMN-driven immunity may represent new venues for intervention in pathological inflammation.

## 1 Introduction

Systemic inflammatory response syndrome (SIRS) is a life-threatening organ dysfunction caused by a dysregulated immune response and is one of the leading worldwide causes of critical illness and mortality (1). One of the cardinal signs of SIRS in addition to confirmed infection is the induction of either fever or hypothermia (2). Interestingly, prevailing dogma indicates that fever manifestation during sepsis is likely to be advantageous for the survival of patients; in contrast hypothermia is associated with increased mortality (3–10). Fever promotes the defense strategies of the immune system, while hypothermia is conversely associated with their downregulation and energy conservation (3). Fever modulates innate immune function by enhancing proliferation, chemotactic ability, and general reactivity of the most abundant white blood cells in the circulation - polymorphonuclear leukocytes (PMNs), comprised mainly of neutrophils (11, 12). To fight and clear potential pathogens, they employ various strategies including phagocytosis, degranulation, and the formation of neutrophil extracellular traps (NETs) (13).

NETs are large, extracellular, web-like structures composed of nuclear and mitochondrial DNA that form a scaffold for histones, neutrophil elastase, myeloperoxidase (MPO), calprotectin, cathelicidin, and other defensins to trap and kill bacteria, important defensive mechanisms in homeostasis and in disease; *e.g.*, sepsis (13–15). *In vivo*, NETs are cleared from the circulation through the action of nucleases and macrophage phagocytosis (16, 17). When left undegraded, NETs that remain in the circulation can promote intravascular coagulation, endothelial tissue damage, and further inflammation, thus contributing to disease progression (18–20). The initiation of a cytokine storm may render the initial infectious insult redundant as immune system also responds to sterile stimuli originating from damaged cells. This together leads to an aberrant immune activation and a lack of response to antibiotic administration as seen in sepsis. Reactivity of CD16+ PMNs to both infectious and sterile stimuli is likely to play a central role in the clinical outcome in conditions including SIRS and sepsis (21). There is, therefore, an urgent need to better understand the mechanism(s) that lead to their activation.

To date, there is limited information on the reactivity of PMNs in fever and hypothermia. Only one study has previously reported the impact of fever-range hyperthermia on *Pseudomonas aeruginosa*-induced NETs formation (22). We extend those initial observations and report modulation of NETs formation in response to gram-positive *Staphylococcus aureus (S. aureus)*, gram-negative *Escherichia coli (E. coli)* and sterile (mitochondria) stimuli at varying temperatures representative of hypothermia, hyperthermia and extreme hyperthermia. The impact of temperature on the phenotype of PMNs and NETs stability, an important aspect of their clearance was also investigated. Our study offers new insights into NET homeostasis in response to temperature; features that may offer new targets for improved therapeutic interventions.

## 2 Methods

### 2.1 Blood collection and PMNs isolation

Healthy adult donors volunteered and consented to the blood collection under ethical approval given by the Ethics Committee of the University Hospital Bratislava (workplace Ruzinov under number EK 218/2020). Blood was collected by venous puncture using BD Vacutainer^®^ blood collection set into 10 ml BD Vacutainer^®^ Heparin Tubes (367878, Becton Dickinson, Plymouth, UK). Whole blood was used for PMNs isolation and *ex vivo* NETs formation assay or centrifuged at 1600g for the collection of plasma. Human PMNs were isolated by 1-Step Polymorphs (AN221725, Accurate Chemical & Scientific Corp, Carle Place, NY, USA). The procedure was done according to the manufacturer protocol with adaptations published elsewhere (23). Finally, PMNs were resuspended in 1 ml of phenol-red free Gibco™ RPMI 1640 Medium (11835030, Paisley, UK) supplemented with 10% FBS (P30-3306, PAN-Biotech, Aidenbach, Germany) medium and counted with LUNA-II™ Automated Cell Counter (LUNA-II, Logos Biosystems, Villeneuve d’Ascq, France).

### 2.2 Cultivation of bacteria

*E. coli* CFT073 (ATCC 700928) and *S. aureus* subspecies *aureus* Rosenbach (NCTC 8530) were inoculated from frozen stocks into LB Broth (L3022, Sigma, Saint-Louis, MO, USA) and incubated overnight in aerobic conditions at 37°C. Next, bacteria were centrifuged at 5000g for 5 minutes and resuspended in sterile PBS (BR0095, Canvax Reagents SL, Valladolid, Spain). Bacterial quantification was determined by optical density (O.D.) at 600 nm using a spectrophotometer (Eppendorf, Germany), where O.D. 1 equaled 8 x 10^8^/ml of colony forming units (CFU) for *E. coli* CFT073 and 7.8 x 10^8^/ml for *S. aureus*. We also verified that an increase or decrease in temperature in the range of 35-42°C did not affect the growth of bacteria (**Supplementary Figure S1**).

### 2.3 Isolation of mitochondria

Previous work from our laboratory has identified human placental tissue as a rich source of mitochondria, which we routinely employ for *ex-vivo* studies (EK 218/2020) (24). Mitochondria were prepared as follows: 100 mg tissue aliquots were dissected with a scalpel and transferred to the Dounce hand homogenizer in 1ml of ice-cold STE buffer (250 mM saccharose, 2 mM EGTA, 5 mM Tris-HCl, pH 7.4) and mechanically homogenized. The whole homogenate was centrifuged at 500g for 3 minutes at 4°C, supernatant was collected and centrifuged again at 8000g for 10 minutes at 4°C. The pellet was resuspended in fresh STE buffer and the whole process was repeated 3 times prior to collection of mitochondria.

Mitochondria purity and quantity were assessed by qPCR using primers designed to amplify part of the D-loop hypervariable region of mitochondrial DNA (forward: 5′-CATAAAAACCCAATCCACATCA-3′, reverse: 5′-GAGGGGTGGCTTTGGAGT-3′) and part of the human globin gene to quantify nuclear DNA (forward: 5′-GCTTCTGACACAACTGTGTTCACTAGC-3′; reverse: 5′CACCAACTTCATCCACGTTCACC-3′). qPCR was performed on the QTower3 (Analytik Jena GmbH, Jena, Germany) using the Advanced Universal SYBR Green Supermix (10000076382, Bio-Rad, Hercules, CA, USA). We routinely isolated ~1 x 10^9^/ml mitochondria from 100 mg of placental tissue.

### 2.4 *In vitro* NETs isolation and quantification

To isolate NETs, a modified version of a previously published protocol was used (25). Briefly, 1x 10^7^/ml isolated PMNs were plated in a 10 cm tissue culture dish (83.3902, SARSTEDT AG & Co., Nümbrecht, Germany) in phenol-red free Gibco™ RPMI 1640 Medium (11835030, Paisley, UK) supplemented with 10% (v/v) FBS (P30-3306, PAN-Biotech, Aidenbach, Germany). PMNs were then exposed to an established NETs inducer 1 µM phorbol-12-myristate-13-acetate (PMA) (P8139-1MG, Saint-Louis, MO, USA) for 3 hours in the 35-42°C temperature range. After the incubation, supernatant was gently aspirated and discarded and NETs that remained attached to the dish were harvested by repeated washing with 10 ml of ice-cold PBS (BR0095, Canvax Reagents SL, Valladolid, Spain). Suspension containing NETs was then first centrifuged at 400g for 10 minutes at 4°C to pellet debris (dead cells) and NETs-rich supernatant was then centrifuged again at 20000g for 10 minutes at 4°C to pellet NETs. NETs-rich pellet was finally resuspended in several aliquots with 100 µl PBS and stored at -80°C until required. NETs induction by PMA was independently verified by fluorescence microscopy (**Supplementary Figure S2A**) and isolated NETs identified by anti-MPO-DNA ELISA (**Supplementary Figure S2B**). NETs were quantified according to DNA content. Briefly, DNA from NETs was isolated using a QIAamp DNA Mini Blood kit (51104, QIAGEN GmbH, Hilden, Germany) according to the manufacturer protocol. DNA concentration was determined using Invitrogen™, Qubit™ 1X dsDNA High Sensitivity (HS) and Broad Range (BR) Assay Kits (Q33230, Q33211, Invitrogen, Eugene, OR, USA).

### 2.5 Quantification of MPO-DNA complexes

MPO-DNA complexes were quantified according to the protocol by Zuo and colleagues (26). Most of the reagents were taken from the Cell Death Detection ELISA kit (11544675001, Roche, Manheim, Germany); and all incubations except for antibody coating were done at room temperature (RT). In short, a high-binding 96-well plate (Costar, Corning, NY, USA) was coated overnight at 4°C with 1 µg/ml of anti-human MPO antibody (0400-0002, Bio-Rad, Hercules, CA, USA) and then blocked with 4% bovine serum albumin (Merck, Saint-Louis, MO, USA) in PBS with 0.05% Tween-20 (AppliChem GmbH, Darmstadt, Germany) for 2 hours at RT. The plate was washed 5 times and incubated for 90 minutes with isolated NETs that were quantified according to their DNA content, washed again and incubated for another 90 minutes with 10× HRP conjugated anti-DNA antibody diluted 100 times. Plate was washed 5 times at the end of the last incubation and developed with 3,3′,5,5′-Tetramethylbenzidine substrate Single Solution (Life Technologies Corporation, Carlsbad, CA, USA) followed by a 2N sulfuric acid Stop Solution (ThermoFisher Scientific, Waltham, MA, USA). Absorbance was measured at 450 nm using a Synergy H4 Hybrid Reader (BioTek, Santa Clara, CA, USA).

### 2.6 Detection of NETs by fluorescence microscopy

1x10^6^ cells/ml PMNs were seeded onto 0.001% poly-L-lysine (3438-100-01, R&D systems, Minneapolis, MN, USA) coated coverslips in phenol-red free Gibco™ RPMI 1640 Medium (11835030, Paisley, UK) supplemented with 10% FBS (P30-3306, PAN-Biotech, Aidenbach, Germany). Control PMNs were left unstimulated, whilst remaining were treated with *E. coli* (multiplicity of infection (MOI) 20), *S. aureus*, (MOI 20), mitochondria (MOI 20) or 100 nM PMA for 3 hours at 37°C and 5% CO_2_. Post-incubation, cells were fixed and stained, with all steps performed at RT in the dark. Fixation and permeabilization was done by the addition of 2% paraformaldehyde for 10 minutes, followed by 0.05% Triton-X100 (T8787-100ML, Sigma, Saint-Louis, MO, USA) for 15 minutes. Next, 1 hour incubation in the blocking buffer consisting of 5% FBS in PBS (BR0095, Canvax Reagents SL, Valladolid, Spain) was conducted. For citrullinated histone H3 staining, fixed cells were incubated for 2 hours with 0.5 µg/100 µl of primary anti-Histone H3 (citrulline R17) antibody (ab219407, Abcam, Cambridge, UK) followed by 1 hour of a 0.5 µg/100 µl of secondary PE Donkey anti-rabbit IgG (minimal x-reactivity) antibody (406421, Biolegend, San Diego, CA, USA). Coverslips were then gently washed with PBS and stained for DNA with 200 nM SYTOX™ Green Nucleic Acid Stain (S7020, Invitrogen, Eugene, OR, USA) for 15 minutes. Following staining, coverslips were mounted with Fluorescent Mounting Medium (4866-20, Trevigen, R&D systems, Minneapolis, MN, USA) and images were collected with an Axiolab 5 fluorescence microscope (Zeiss, Jena, Germany) using Filter Sets 43 and 44 (Zeiss, Jena, Germany).

### 2.7 Analysis of NETs degradation in plasma

50 ng/ml of NETs were stained with 200 nM SYTOX™ Green Nucleic Acid Stain (S7020, Invitrogen, Eugene, OR, USA) and incubated in either plasma of a corresponding donor or PBS (BR0095, Canvax Reagents SL, Valladolid, Spain) in a thermal gradient ranging from 35-42°C using a real-time PCR machine QTower3 (Analytik Jena GmbH, Jena, Germany). Fluorescence signal was read on a FAM channel at 10 minutes intervals over a period of 6 hours. Effect of exogenously administered DNase I was analyzed by adding RNase-Free DNase I (79254, QIAGEN GmbH, Helden, Germany) to a final concentration 2.5 KU/ml. Quantity of undegraded NETs was expressed in % and calculated from the fluorescence signal of a sample normalized to the signal of NETs in PBS.

### 2.8 Quantification of PMN DNA release

5 x 10^4^/ml PMNs were incubated in 100 µl of complete RPMI medium supplemented with 200 nM SYTOX™ Green Nucleic Acid Stain (S7020, Invitrogen, Eugene, OR, USA) in the presence of *E. coli, S. aureus*, mitochondria (all MOI 80) or 100 nM PMA. 0. PMNs were lysed with 0.05% Triton X-100 (T8787-100ML, Sigma, Saint-Louis, MO, USA) for quantification of maximum signal. All samples were assayed utilizing real time PCR machine QTower3 (Analytik Jena GmbH, Jena, Germany) for 3 hours at a temperature gradient ranging from 35-42°C. DNA fluorescence was detected on a FAM channel every 10 minutes. Quantity of PMNs releasing DNA was expressed in % of the maximum signal of the sample treated by 0.05% Triton X-100 (T8787-100ML, Sigma, Saint-Louis, MO, USA). For the analysis of DNA release onset, an automatic threshold was used.

### 2.9 Analysis of NETs formation and PMN phenotype in whole blood

100 µl of whole blood was treated with 100 nM PMA or 10 µl of either *E. coli, S. aureus* or mitochondria set to O.D. 1 (600 nm) for 3 hours at 35-42°C. Post-treatment, erythrocytes were lysed using ice-cold isotonic ammonium chloride buffer (150 mM NH_4_Cl, 10 mM KHCO_3_, 0,1 mM EDTA), and leukocytes were centrifuged at 400g for 10 minutes at 4°C, and the pellet was resuspended in FACS buffer (PBS with 1% of FBS). All samples were then stained for 15 minutes at RT in the dark. All antibodies and dyes except primary anti-Histone H3 (citrulline R17) antibody were purchased from Biolegend, San Diego, CA, USA.

Analysis of NETs by flow cytometry was conducted as published with gating verified accordingly (27, 28). Staining was done as follows: 0.1 µg/sample primary rabbit monoclonal anti-Histone H3 (citrulline R17) (ab219407, Abcam, Cambridge, UK) antibody followed by 0.025 µg/sample of secondary Brilliant Violet 510™ Donkey anti-rabbit IgG (406419), in combination with 0.05 µg/sample FITC anti-MPO (347201) and 1 µM DRAQ7™ (424001) were used.

To assess PMNs phenotype, antibody mix used for the detection of NETs was combined with 0.05 µg/sample of anti-human CD16 APC (360706) and either the mix containing 0.04 µg/sample of anti-human antibody CD49d PE/Cyanine 7 (304314) and 0.2 µg/sample PerCP/Cyanine5.5 anti-human formyl peptide receptor 1 (FPR1) (391608) or with 0.025 µg/sample of PE/Cyanine7 Annexin V (640950) to quantify apoptosis. Stained samples were transferred on ice and immediately assayed on a DxFlex flow cytometer (Beckman Coulter, Brea, CA, USA), where data were collected on the appropriate channels. Dynamic gains were set upon previous testing to 100. Samples were analyzed by FCSExpress 6.0 software (*De Novo* Software, Pasadena, CA, USA) purchased from Biolegend, San Diego, CA, USA.

### 2.10 Analysis of phagocytosis

Firstly, all agents were labelled; for this purpose, *E. coli*, *S. aureus* and mitochondria were set to the concentration of 1 x 10^8^/ml in PBS (BR0095, Canvax Reagents SL, Valladolid, Spain). Bacteria were labelled with 1 µM (*E. coli*) or 0.5 µM (*S. aureus*) BacLight™ Red Bacterial Stain (B35001, Invitrogen, Eugene, OR, USA) whilst the mitochondria preparation was exposed to 100 nM MitoView™ Green (70054, Biotium, Fremont, CA, USA). Staining was performed in the dark at 37°C for 30 minutes (*E. coli*) or for 15 minutes (*S. aureus* & mitochondria). All preparations were then washed at 5000g for 5 minutes.

100 µl of whole blood was treated with 10 µl of appropriate stimuli for 30 minutes at 35-42°C. Post-treatment, all cells were washed and subsequently centrifuged to wash away non-ingested bacteria or mitochondria that are not internalized in cells. Erythrocytes were then lysed and stained for 15 minutes at RT in the dark for flow cytometric analysis.

To assess PMN phenotype, samples were stained with the mix of 0.05 µg/sample of anti-human CD16 APC (360706), 0.1 µg/sample of APC anti-mouse/human CD11b (101212) and 1 µM DRAQ7™ (424001). Stained samples were transferred on ice (to inhibit phagocytosis) and immediately assayed on a DxFlex flow cytometer (Beckman Coulter, Brea, CA, USA), where data were collected in the appropriate channels. Dynamic gains were set to 100. Samples were analyzed by FCSExpress 6.0 software (*De Novo* Software, Pasadena, CA, USA) purchased from Biolegend, San Diego, CA, USA.

### 2.11 Nuclease activity of plasma

For the quantification of plasma nuclease activity, a customized short PE-labelled DNA oligonucleotide probe was used, as reported previously (29). The DNase probe was added to plasma at a final concentration of 0.25 μM and incubated for 1 hour in the temperature range 35-42°C. Nuclease activity was expressed in KU/ml and the calibration was done according to the activity of DNase I (79254, QIAGEN GmbH, Hilden, Germany) at 37°C in a DNase I buffer (0.1 mM CaCl_2_, 0.3 mM MgCl_2_, 10 mM Tris-HCl, pH= 7.4).

### 2.12 Cytokine analysis

1 ml of whole blood was treated with 100 µl *E. coli, S. aureus* or mitochondria set to O.D. 1 (600 nm) or 100nM PMA for 3 hours at 35°C-42°C. Post-stimulation, the sample was centrifuged at 1600g at 4°C and plasma collected. The concentrations of cytokines (IL-1β, IFN-α2, IFN-γ, TNF-α, MCP-1, IL-6, IL-8, IL-10, IL-12p70, IL-17A, IL-18, IL-23, and IL-33) were determined using the LEGENDplex™ Human Inflammation Panel (13-plex) in V-bottom plates (Biolegend, San Diego, CA, USA) and measured on a DxFlex cytometer (Beckman Coulter, Brea, CA, USA) according to the manufacturer’s instructions.

### 2.13 TLR9-mediated NF-κB activation

Human Toll-like receptor 9 (TLR9)-expressing HEK-Dual™ hTLR9 (NF/IL8) reporter cells (hkd-htlr9ni, Invivogen, San Diego, CA, USA) were selected with antibiotics according to manufacturer’s instructions and cultured in DMEM (11965092, Paisley, UK), supplemented with heat inactivated 10% (v/v) FBS (P30-3306, PAN-Biotech, Aidenbach, Germany) at 37°C in 5% CO_2_. 1 x 10^5^ cells/well were stimulated with 100 pg/ml of NETs for 20 hours. ODN 2006 (tlrl-2006, Invivogen, San Diego, CA, USA) was used as a positive control. ODN 2006 (5’-TCGTCGTTTTGTCGTTTTGTCGTT-3’) is a type B CpG oligonucleotide with a phosphorothioate backbone that contains unmethylated CpG dinucleotides and is a potent activator of TLR9. Cell culture supernatants were harvested and analyzed for NF-κB reporter activity according to the instructions provided. Absorbance was measured using a Synergy H4 Hybrid Reader (BioTek, USA). Raw data can be found in the supplement (**Supplementary File 1**).

### 2.14 Statistical analysis

Statistical analysis was performed using GraphPad Prism v8.00 for Windows (GraphPad Software, La Jolla, CA, USA). TLR9 activation by NETs was analyzed by the Kruskal-Wallis test. Pearson’s correlation coefficient was calculated for the correlation between MPO-DNA complexes and DNA concentration, as well as plasma nuclease activity and temperature. All other results were evaluated using two-factor ANOVA followed by Dunnett’s multiplecomparisons test. Data are presented as a mean and standard deviation (SD). P values < 0.05 were considered statistically significant.

## 3 Results

### 3.1 Temperature modulates intensity and onset of PMN DNA release induced by infectious and sterile inflammatory stimuli

**Figure 1:**
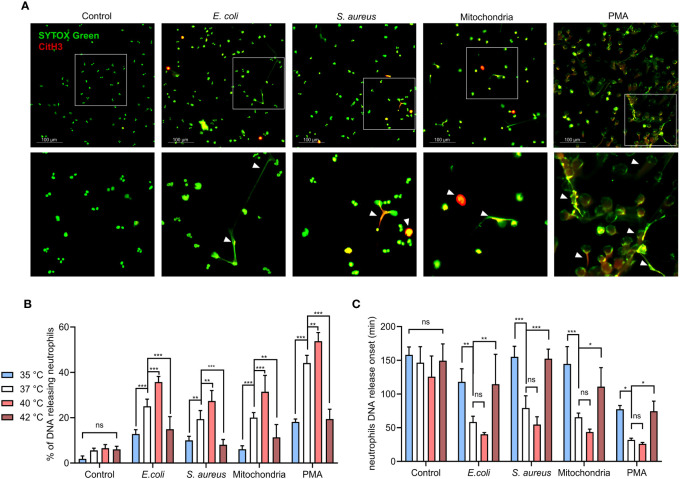
Effect of temperature on magnitude and onset of PMNS DNA release. **(A)** Representative images of NETs formation in response to *E. coli*, *S. aureus*), mitochondria and PMA after 3 hours incubation at 37°C. NETs are identified according to positivity for citrullinated histone H3 and SYTOX GreenTM staining. **(B)** DNA release of isolated PMNs in response to, *E. coli* (MOI 80), *S. aureus* (MOI 80), mitochondria (MOI 80) and 100 nM PMA incubated for 3 hours at temperatures of 35°C, 37°C, 40°C and 42°C, quantified according to maximum signal of lysed neutrophils. **(C)** Comparison of the DNA release onset at selected temperatures of 35°C, 37°C, 40°C and 42°C in response to the stimuli described above. Difference in onset of NETs formation at 37°C vs. 40°C; control (p = 0.42), E. coli (p = 0.528), *S. Aureus* (p = 0.291), mitochondria (p = 0.374), PMA (p = 0.967). The data presented are means ±SD of 4 independent experiments performed in duplicates. * p < 0.05, ** p < 0.01, *** p < 0.001; two-factor ANOVA followed by Dunnett’s multiple comparisons test.

To model the response of neutrophils to common pathogens that initiate sepsis and to tissue damage that occurs later in its pathology, we have treated isolated PMNs with representative strains of gram-negative (*E. coli*, CFT073) and gram-positive (*S. aureus*, NCTC 8530) bacteria and mitochondria (30, 31). First, we used fluorescence microscopy to verify that both, infectious (*E. coli, S. aureus*) and sterile (mitochondria) stimuli, induce the formation of NETs in isolated PMNs. NETs were identified by the presence of histone H3 citrullination and morphology of the released extracellular DNA. NETs formation was greatest in response to PMA, followed by *E. coli*, mitochondria and *S. aureus* (**Figure 1A**).

To establish how temperature affects the dynamics of PMNs DNA release, isolated PMNs were incubated in the presence of *E. coli* (MOI 80), *S. aureus* (MOI 80) or mitochondria (MOI 80) at temperatures ranging from 35.3-42.2°C (real-time PCR machine). DNA release was quantified at 10 minutes intervals for 3 hours, 100 nM PMA stimulation served as a positive control. PMN DNA is released during cell death, including the formation of NETs. As PMNs employ multiple antimicrobial defense mechanisms each with varying kinetics, it was important to note that NETs formation exhibits slow kinetics (1 to 2 hours). Next, we wanted to elucidate how temperature may affect the kinetics of NETs formation. Although non-specific for a type of cell death, this allowed for the selection of temperatures that will be further investigated in the analysis of NETs formation. We observed no statistically significant effect of temperature on DNA release in the control, unstimulated PMNs, interestingly, percentage of cells releasing DNA concurrently increased in response to all stimuli, showing a maximal response at 40°C, whilst the response at 42.2°C remained impaired (**Supplementary Figure S3A**). From these results, we selected the most clinically relevant values as reference temperatures for further comparisons: hypothermia represented by 35°C, normothermia by 37°C, febrile range fever at 40°C and 42°C for critical hyperthermia (**Figure 1B**). To investigate whether temperature affects the dynamics of DNA release, we also recorded the onset of DNA release. It was interesting to note that DNA release onset was delayed at 35.3°C and 42.2°C when compared to normothermia (**Figure 1C**). No statistically significant difference in DNA release in response to all treatments were observed between 36-41°C, although a modest trend towards earlier DNA release with increasing temperature was noted (**Supplementary Figure S3C**). To summarize, temperature positively affects the quantity of the released PMN DNA in the range between 36-41°C in response to all stimuli. The extreme temperatures of 35.3°C and 42.2°C mediated a delay in the onset and in the magnitude of DNA release.

### 3.2 Release of PMN DNA in response to infectious and sterile inflammatory stimuli is maximal at 40°C

When compared to 37°C, 40°C exposure caused increased DNA release in response to all of the tested stimuli by 8-11% (p < 0.01 for all). Conversely, decreased DNA release was observed for all of the treated groups at both 35°C and 42°C when compared to 37°C. At 35°C, DNA release in comparison to normothermia was decreased by 9-14% for *E. coli*, *S. aureus* and mitochondria (p < 0.01 for all) and 26% for PMA (p < 0.001). At 42°C, we have observed a 9-11% decrease for *E. coli*, *S. aureus* and mitochondria (p < 0.01 for all) and 25% for PMA (p<0.001) (**Figure 1B**).

Temperatures at both extremes of 35°C and 42°C also delayed onset of DNA release. At 37°C, *E. coli* induced DNA release at ~58 minutes, mitochondria at ~65 minutes and *S. aureus* at ~79 minutes. PMA induced DNA release the fastest, with the onset being at ~31 minutes. When compared to 37°C, 40°C promoted onset by 18-24 minutes for *E. coli*, *S. aureus* and mitochondria and ~5 minutes for PMA, but none of the differences were statistically significant. At 35°C, DNA release when compared to 37°C was delayed by 59-79 minutes for *E. coli*, *S. aureus* and mitochondria (p < 0.01 for all) and by ~45 minutes for PMA (p < 0.05). Finally, incubation at 42°C delayed DNA release in comparison to 37°C by 42-73 minutes for *E. coli*, *S. aureus* and mitochondria (p < 0.05 for all) and ~42 minutes for PMA (p < 0.05) (**Figure 1C**). When analyzed from the perspective of specific temperatures, treatment with *E. coli*, *S. aureus* and mitochondria did not show any significant difference in the magnitude of induced DNA release nor its onset (**Supplementary Figures S3B, D**). Collectively, our observations suggest that in addition to the stimuli, temperature is also a determinant of NETs formation.

### 3.3 Temperature modulates NETs formation but not PMNs apoptosis when induced *ex vivo*

Response of PMNs to either infection or sterile inflammation *in vivo* is modulated by the microenvironment milieu, which will constitute other immune/non-immune cells and cytokine flux. *Ex-vivo* analysis in whole blood offers a step closer to an *in-vivo* environment, a procedure previously employed by investigators studying NETs formation (32–35). We utilized established markers of NETs (MPO, citrullinated histone H3 and extracellular DNA) to quantify CD16+ PMNs derived NETs in whole blood via flow cytometry.

Due to the sticky nature of NETs, exclusion of cell doublets was not conducted. In addition, we also verified the potential presence of NETs outside the gate for PMNs; minimal events of double-positivity (citrullinated histone H3 and MPO) were recorded in the debris or in the lymphocyte/monocyte population. We found minimal change in cellular granularity in PMNs treated with either bacteria or mitochondria, following a modified protocol from a previous publication (28).

**Figure 2:**
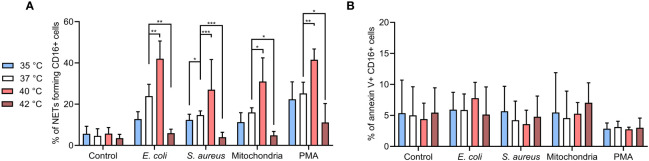
**(A)** % of NETs forming CD16+ PMNs in blood incubated for 3 hours at 35°C, 37°C, 40°C and 42°C treated with, *E. coli* (MOI 80), *S. aureus* (MOI 80) and mitochondria (MOI 80) and 100 nM PMA. MOI was calculated according to the number of PMNs of the corresponding sample. **(B)** % of apoptotic CD16+ PMNs in blood incubated as described above. Gating strategy can be found in the **Supplementary Figure S5**. The data presented are means ±SD of 4 independent experiments performed in duplicates. * p<0.05, ** p<0.01, *** p < 0.001; two-factor ANOVA followed by Dunnett’s multiple comparisons test.

For CD16+ PMNs, mean NETs % in control cells were ~5% at all temperatures. Briefly, all agents induced NETs formation at 37°C, *E. coli* and PMA induced more NETs (23.9% and 25.2%, respectively) when compared to *S. aureus* or mitochondria (14.7% and 16%, respectively). Incubation at 40°C further increased NETs formation by 12.3-18.1% when compared to 37°C (p < 0.05 for all). For CD16+ PMNs incubated at 35°C, relative NETs formation when compared to 37°C decreased by 2.4-4.7% for *S. aureus*, mitochondria and PMA. Interestingly, *E. coli* caused ~11% reduction, the most significant impairment amongst the agents tested (p < 0.05). Of note, impairment of NETs formation at 42°C was greater in magnitude (10.7-18%) and importantly reached statistical significance for all stimuli (p < 0.05 for all) (**Figure 2A**).

To clarify the impact of temperature on apoptosis *versus* NETs formation, we analyzed the CD16+ PMNs for annexin V positivity. Overall, <10% of cells showed apoptosis in all samples with no differences regardless of the treatment or temperature (**Figure 2B**). Collectively, our observations support the notion that temperature is a key determinant of PMNs NETs formation, whilst PMN apoptosis showed less propensity for temperature dependency in the experimental time-frame tested in the present study.

### 3.4 CD16+ PMNs expressing CD49d form a small population that is responsible for the majority of NETs formation

In homeostasis, ~10-15% of CD16+ PMNs express CD49d, an important mediator involved in neutrophil adhesion and migration and its expression increases as neutrophils age (36, 37). It is noteworthy that a dramatic upregulation of CD49d expression has been reported in septic blood neutrophils compared to healthy donors (38). Aged neutrophils are considered to be more reactive and show a higher capacity to form NETs (36). The impact of temperature on CD16+ CD49D+/- populations remains unstudied.

**Figure 3:**
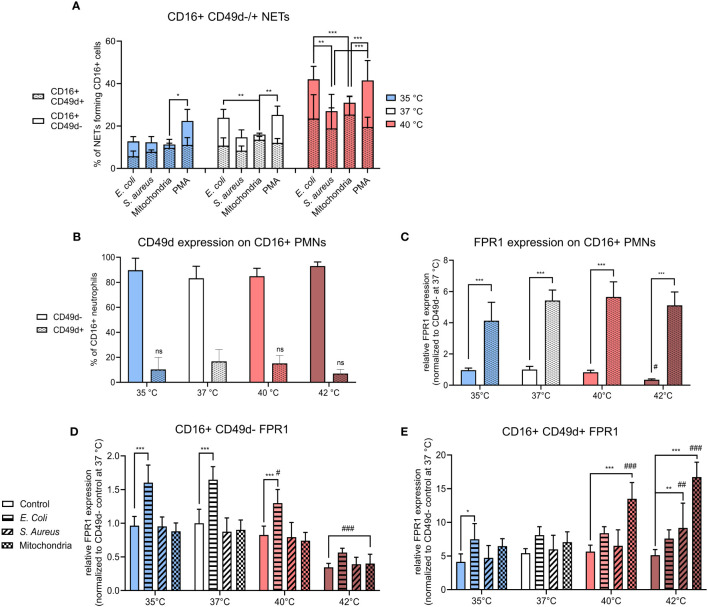
**(A)** % of CD16+ CD49d-/+ NETs forming cells from all CD16+ PMNs forming NETs incubated at 35°C, 37°C and 40°C and treated with *E. coli* (MOI 80), *S. aureus* (MOI 80) and mitochondria (MOI 80). **(B)** Relative counts of CD49d-/+ CD16+ PMNs in control samples. **(C)** Relative expression of FPR1 in control CD16+ PMNs according to CD49d expression status normalized to the expression of control CD16+ C49d- PMNs at 37°C. **(D)** Relative expression of FPR1 in stimulated CD16+ CD49d- PMNs normalized to the expression of control CD16+ CD49d- PMNs at 37°C. **(E)** Relative expression of FPR1 in stimulated CD16+ CD49d+ PMNSs normalized to the expression of control CD16+ CD49d- PMNs at 37°C. Gating strategy can be found in the **Supplementary Figure S6**. The data presented are means ±SD of 4 independent experiments. * p < 0.05, **/## p < 0.01, ***/### p < 0.001; # is difference vs. respective treatment at 37°C; two-factor ANOVA followed by Dunnett’s multiple comparisons test.

We found that CD16+ CD49d+ PMNs were responsible for ~45-85% of all NETs formation, irrespective of treatment or temperature (**Figure 3A**). The contribution of the majority (CD16+CD49d- PMNs) to NET formation was also followed. At 35°C, the response of CD16+ CD49d- PMNs to PMA was 11% higher when compared to mitochondria stimulation (p < 0.05); at 37°C their contribution to NET formation was 10% higher to both *E. coli* and PMA than to mitochondria (p < 0.01 for both). At 40°C, CD16+ CD49d- PMNs responded with ~11-14% higher NETs formation to *E. coli* than to *S. aureus* or mitochondria (p < 0.01 for both). Similarly, response to PMA was higher by 14-17% than to *S. aureus* and mitochondria (p < 0.001 for both) (**Figure 3A**). As no increase in NETs formation was seen at 42°C, we have not included any of those samples in the comparison.

In summary, CD16+ CD49d+ PMNs comprised only between ~7-16% of all control CD16+ PMNs. Temperature has no effect on CD49d expression on CD16+ PMNs, however, a trend of lower CD49d expression was seen at 42°C (**Figure 3B**). Despite being a small component, this subset was responsible for majority of the NETs formation in response to all stimuli. It is worth noting that in response to more potent NET inducers such as *E. coli* and PMA, contribution of the CD49d- PMNs compartment showed a trend for greater input towards NET formation between 35-40°C.

### 3.5 CD16+ CD49d+ PMNs have higher FPR1 expression than CD16+ CD49d- PMNs and temperature modulates FPR1 expression in both subsets differently

To further examine the phenotype of CD16+ CD49d-/+PMNs, we have also analyzed relative expression of FPR1 that is implicated in pathogen recognition during infection. Primary function of FPR1 is the recognition of formylated peptides present on the surface of both bacteria and mitochondria, upon engagement the receptor-ligand complex is internalized, promoting PMN migration and antimicrobial immunity (21, 39, 40). Since PMA is an artificial stimulus that does not interact with this receptor, we have analyzedits expression only in response to *E. coli*, *S. aureus* and mitochondria.

When normalized to control CD16+ CD49d- PMNs at 37°C, FPR1 expression was 5-times higher in control CD16+ CD49d+ PMNs at all temperatures (p < 0.001 for all). Compared to 37°C, incubation at 42°C resulted in a 3-times decrease in the relative expression of FPR1 on control CD16+ CD49d- PMNs, but the difference was not statistically significant. FPR1 expression was not altered in CD16+ CD49d+ PMNs (**Figure 3C**). Next, we examined treatment-induced change in FPR1 expression in both CD16+ CD49d- and CD49d+ PMNs in response to temperature.

In CD16+ CD49d- PMNs, only treatment with *E. coli* resulted in a 1.6-times increase in expression of FPR1 at 35-40°C when compared to respective controls (p < 0.001 for all). When the effect of temperature within each treatment was studied, we observed a 1.8-3-times decrease in expression of FPR1 at 42°C *vs*. 37°C in all CD16+ CD49d- PMNs (p < 0.001 for all). Additionally, when compared to 37°C, increasing the temperature to 40°C resulted in a 1.3-times decrease in FPR1 expression for CD16+ CD49d- PMNs treated with *E. coli* (p < 0.05) (**Figure 3D**).

For CD16+ CD49d+ PMNs, however, treatment with *E. coli* resulted in a 1.8-times increase in FPR1 expression at 35°C (p < 0.05) and treatment with *S. aureus* resulted in a 1.8-times increase at 42°C (p < 0.01) when compared to respective controls. Mitochondria induced FPR1 expression 2.4-3.3 fold at 40°C and 42°C (p < 0.001 for both) when compared to their respective controls. We have then further examined the effect of temperature on FPR1 expression within each treatment and found when compared to 37°C, mitochondria induced a 1.9-2.4-times increase in FPR1 expression at 40°C and 42°C (p < 0.001 for both) and *S. aureus* 1.5-times increase at 42°C (p < 0.01) (**Figure 3E**).

To summarize, FPR1 expression was ~5-fold greater in CD49d+ PMNs, when compared to CD49d- PMNs and this profile did not change within the range of 35-40°C in untreated cells. Temperature, however, did exhibit a differential effect on FPR1 expression in response to infectious and sterile stimuli in CD49d- versus CD49d+ PMNs. In CD49d- PMNs, FPR1 expression increased in response to *E. coli* between 35-37°C, the effect decreasing at higher temperatures. CD49d+ PMNs increased FPR1 expression mostly in response to mitochondria at temperatures of 40°C and 42°C, but not at lower temperatures. *S. aureus* did not induce a change in FPR1 expression except at 42°C, where an upregulation was seen on CD49d+ PMNs. Together, the data suggests that a high FPR1 expression is associated with the CD49d+ PMNs phenotype and is further differently modulated in CD49d+ and CD49d- compartments dependent largely on the nature of the stimuli with temperature playing a contributory role.

### 3.6 Increasing temperature positively affects CD16+ PMNs phagocytosis

**Figure 4:**
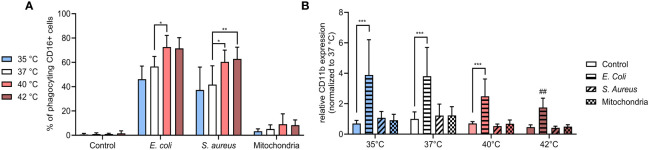
**(A)** % of CD16+ PMNs phagocyting *E. coli, S. aureus* and mitochondria at all temperatures. **(B)** Relative expression of CD11b normalized to the expression of control CD16+ PMNs at 37°C. Gating strategy can be found in the **Supplementary Figure S7**. The data presented are means ±SD of 4 independent experiments. * p<0.05, ** p<0.01, *** p < 0.001; # is difference vs. respective treatment at 37°C; two-factor ANOVA followed by Dunnett’s multiple comparisons test.

As prevailing dogma indicates that the choice to either phagocytose or form NETs can be dichotomic, we next investigated how temperature affects PMN phagocytosis (41). CD16+ PMNs exhibited increased phagocytosis towards both *E. coli* and *S. aureus* at 40°C and 42°C when compared to 37°C. Specifically, phagocytosis of *E. coli* increased in CD16+ PMNs increased from 57% at 37°C to 73% at 40°C (p < 0.05) and phagocytosis of *S. aureus* increased from 42% at 37°C to 60-63% at 40°C and 42°C (p < 0.05 for both) (**Figure 4A**). We found minimal phagocytosis of mitochondria, with no observable differences at varying temperatures.

Next, we analyzed the relative expression of CD11b which exhibits modest expression on the surface of unstimulated PMNs but increases upon exposure to bacterial pathogens such as *E. coli* (42, 43). CD11b is a member of β integrin family and mediates neutrophil diapedesis, adhesion to endothelium and is also involved in phagocytosis of complement opsonized bacteria. Studies examining change in CD11b expression on neutrophils from sepsis patients in comparison to healthy controls to date has yielded conflicting information (44–49). We observed that only stimulation with *E. coli* resulted in a relative increase of CD11b expression and this change was temperature dependent. When compared to the control, *E. coli*-induced expression increased ~3.5-5.5 fold between 35-40°C (p < 0.001 for all), with a declining trend observed with an increase in temperature. At 42°C, CD11b expression was increased 3.8-times, however, the difference was not significant and when compared to the induction at 37°C, was relatively decreased 2.2-times (p < 0.01) (**Figure 4B**). All other treatments had no impact on CD11b expression in any temperature.

Our findings show that CD16+ PMNs predominantly phagocytize bacteria, but not mitochondria and the process of phagocytosis is upregulated at 40°C and 42°C. The fact that treatment at 40°C resulted in both increased phagocytosis and NETs formation suggests these two processes may not be mutually exclusive. In contrast to the trend observed in % of phagocyting cells, surface expression of CD11b was in response to *E. coli* upregulated the most at 35°C and got lower at higher temperatures.

### 3.7 Bacteria induce pro-inflammatory cytokine release at all temperatures except for 42°C and mitochondria only at 37°C

**Figure 5:**
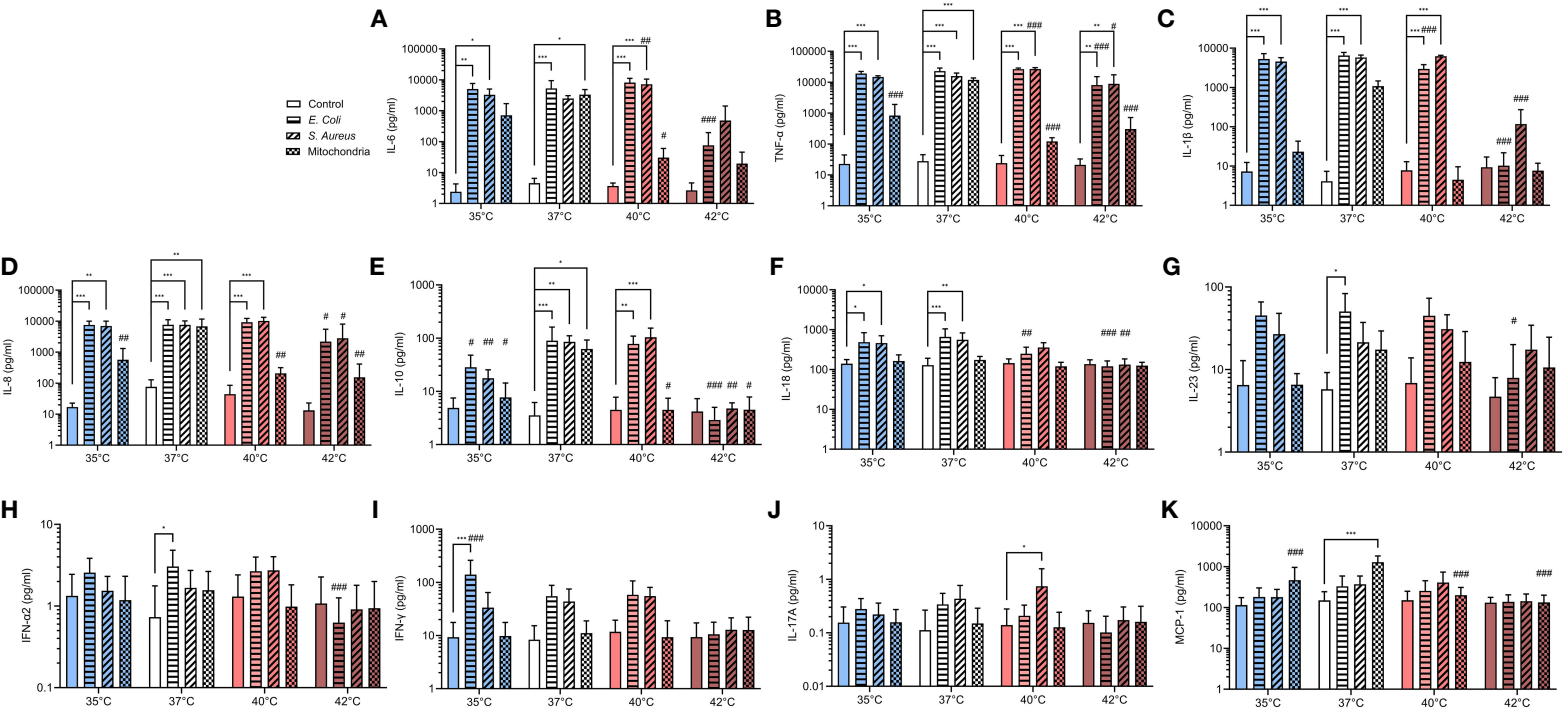
Pro- and anti-inflammatory cytokines release in response to infectious and sterile stimuli at all temperatures **(A-K)** Plasma concentration of IL-6, TNF-α, IL-1β, IL-8, IL-10, IL-17A, IL-18, IL-23, MCP-1, IFN-α2 and IFN-γ in response to *ex vivo* stimulation of blood by *E. coli*, *S. aureus* and mitochondria at all temperatures. The data presented are means ± SD of 4 independent experiments. *p < 0.05, **/## p < 0.01, ***/### p < 0.001; # is difference *vs*. respective treatment at 37°C; two-factor ANOVA followed by Dunnett’s multiple comparisons test.

Pro-inflammatory cytokine burst is critical for the activation of PMNs in response to stimuli. IL-8 is a potent chemoattractant for neutrophils and also enhances their antimicrobial activity and cytokines such as IL-1β and TNF-α can potentiate NETs formation (50–52). It is also known that during infection, immune system upregulates the concentration of main pyrogenic cytokines such as IL-6, IL-1β and TNF-α that mediate the fever response (53, 54). We have therefore analyzed the profile of several pro- and anti-inflammatory cytokines that were released in response to *E. coli*, *S. aureus* and mitochondria. We observed that between 35-40°C, both *E. coli* and *S. aureus* increased IL-6, IL-1β, TNF-α and IL-8 by 100-1000-times when compared to control (p < 0.05 for all) (**Figures 5A–D**). Mitochondria induced a similar response at 37°C (p < for all), but at both 35°C and 40°C, only a trend was observed, and the increase was not significant. At 42°C, the concentration of IL-6, IL-1β and IL-8 has not changed when compared to control and only the concentration TNF-α increased 1000-times in response to both *E. coli* and *S. aureus* (p < 0.01 for both), but not mitochondria. At 40°C, *S. aureus* was the only stimulus that induced a further 1.7-2.9 increase in concentration of IL-6 and TNF-α when compared to 37°C (p < 0.01 for both). The concentration of the anti-inflammatory cytokine IL-10 was increased 20-times in response to all stimuli at 37°C (p < 0.05 for all) (**Figure 5E**). At 40°C, *E. coli* and *S. aureus*, but not mitochondria, induced a similar response (p < 0.01 for both). No change in IL-10 concentration was observed at 35°C or 42°C in response to any of the stimuli. The concentration of IL-8 increased 3.3-5.1-times in response to both *E. coli* and *S. aureus* at 35°C and 37°C, but not at higher temperatures (p < 0.05 for all) (**Figure 5F**). For the other cytokines, treatment with *E. coli* induced an 4.3-8.7-times increase in the concentration of both IL-23 and IFN-α2 at 37°C and in the case of IFN-γ, 15-times at 35°C (p < for all) (**Figures 5G–I**). *S. aureus* induced the production of IL-17A at 40°C 5.4-times (p < 0.05), albeit the difference is not clinically relevant and treatment with mitochondria resulted in an 8.6-times increase in MCP-1 at 37°C (p < 0.001) (**Figures 5J, K**). No differences in the concentration of IL-12p70 and IL-33 were observed in response to any of the treatments at all temperatures (**Supplementary Figure S4**). Taken together, at all temperatures except for 42°C, both bacteria induced an increase in the concentration of main pyrogenic pro-inflammatory cytokines IL-6, IL-1β and TNF-α as well as IL-8, which mainly regulates the response of polymorphonuclear cells. Mitochondria, on the other hand, induced a response in these cytokines only at 37°C and the response at both higher and lower temperatures was present either as a trend in increase or none at all.

### 3.8 NETs induced at all temperatures are equally potent at activating TLR9-dependent NF-κB signaling

**Figure 6:**
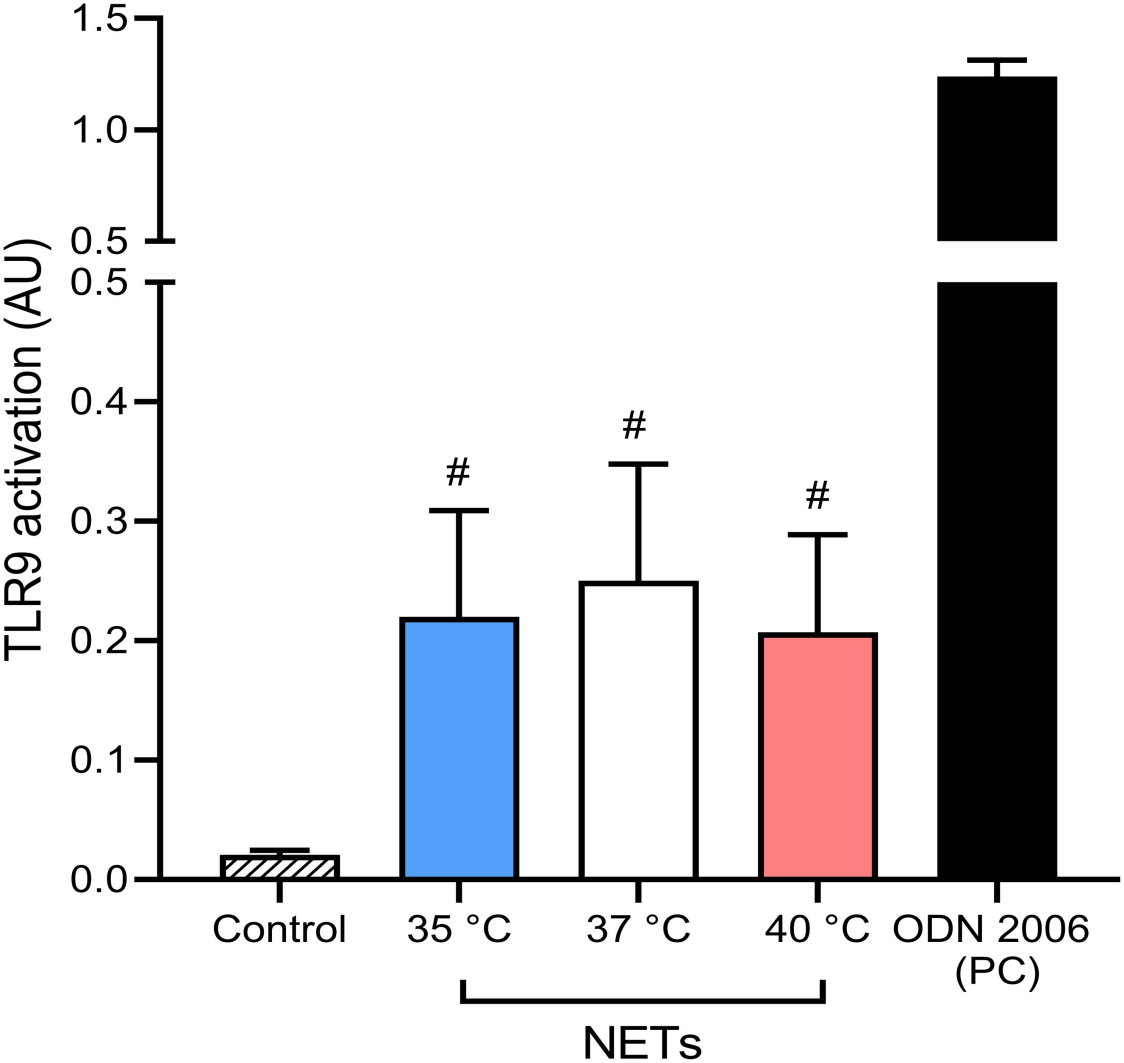
Induction of NF-κB signaling via TLR9 by NETs formed at different temperatures. Induction of TLR9 dependent NF-κB signaling with NETs formed at 35°C, 37°C and 40°C together with 5 µM oligodeoxynucleotides (ODN) 2006 used as a positive control. The data presented are means ± SD of 3 independent experiments assayed in duplicates. # p<0.05 *vs*. negative control; Kruskal-Wallis test.

When not cleared from the circulation, NETs have been described to cause tissue damage and promote inflammation via multiple toll-like receptors, specifically TLR9 (55, 56). These findings support the hypothesis that undegraded NETs formed in response to the initial stimulus may induce the formation of more NETs. It is important to note that TLR9 activation is affected not only by the concentration of the DNA containing unmethylated CpGs but also by the length and the presence of bound proteins to the ligand (57). Since it is currently unknown if temperature affects these properties in NET DNA, we next analyzed the potential of NETs formed at different temperatures to activate NF-κB signaling via TLR9 using an *in vitro* inducible reporter system. All NETs mediated NF-κB activation (p < 0.05 for all), interestingly, no significant difference between the various NETs preparations was recorded (**Figure 6**). Our findings suggest that temperature plays a minimal role in modulating NETs capacity to mediate TLR9 activation.

### 3.9 Higher temperature enhances nuclease activity of plasma and NETs degradation

NETs degradation is pivotal for the resolution of inflammation and is in physiological conditions initially driven by nucleases and later picked up by macrophages (16, 17, 58). Blood plasma has an inherent nuclease and enzymatic activity, and as all enzymatic activity is temperature-dependent (59). We therefore reasoned that fever could enhance NET degradation in plasma. We analyzed whether temperature influences the relative size of DNA in NETs. This was investigated according to the DNA fluorescence pattern of NETs previously detected by flow cytometry (**Supplementary Figure S8**).

**Figure 7:**
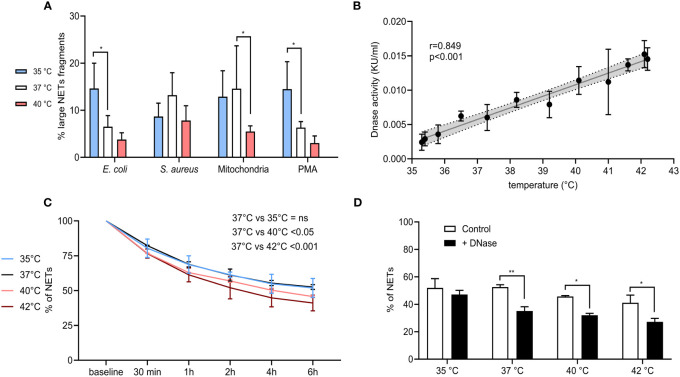
**(A)** % of large NET fragments formed at 35°C, 37°C and 40°C from CD16+ PMNs forming NETs treated with *E. coli* (MOI 80), *S. aureus* (MOI 80) and mitochondria (MOI 80). Gating strategy can be found in the **Supplementary Figure S8**. **(B)** Correlation of plasma nuclease activity and temperature ranging from 35.3-42.2°C. **(C)** Degradation of NETs incubated in plasma for 6 hours at 35°C, 37°C, 40°C and 42°C. **(D)** Degradation of NETs incubated in plasma for 6 hours at 35°C, 37°C, 40°C and 42°C with and without the addition of DNase I at the concentration of 0.25 KU/sample. The data presented are means ±SD of 3 independent experiments assayed in duplicates. * p<0.05, ** p < 0.01; Dunnett’s multiple comparisons test for all experiments except **(B)** which was analyzed by Pearson’s correlation.

In all samples, we recorded the presence of two distinct peaks corresponding to a higher and lower DNA amount that were subsequently quantified and expressed as a percentage of large NETs fragments. For PMA and *E. coli*, NETs formed at 35°C had ~14.5% of large fragments when compared to ~6.5% at 37°C (p < 0.05 for both) and while we detected a decreasing trend in large NETs content induced by both stimuli at 40°C, the difference was not statistically significant. In the case of *S. aureus*-induced NETs, no effect of temperature was seen. Finally, NETs induced by mitochondria at 37°C had 14.5% of large fragments when compared to 5.5% at 40°C (p < 0.05), while no difference was observed between NETs formed at 35°C and 37°C (**Figure 7A**).

Next, we analyzed the endogenous DNase activity of plasma in relation to temperature and observed a strong positive correlation (r=0.859) (p < 0.001) (**Figure 7B**). NETs from isolated PMNs were analyzed for the capacity of plasma to degrade them at all tested temperatures. Incubation of NETs in self-plasma for 6 hours at 37°C resulted in their degradation to almost 50%, when degradation rate reached a plateau and further incubation had no effect. When compared to incubation at 37°C, a lower temperature of 35°C did not alter NETs degradation, however incubation of NETs at 40°C and 42°C further enhanced their degradation, specifically by 6% at 40°C and by 10% at 42°C (p < 0.05 for both) (**Figure 7C**). Administration of exogenous DNase I at 37-42°C increased NETs degradation by 13.7-19.9% (p < 0.05 for all), no change at 35°C was observed (**Figure 7D**). To summarize, degradation of NETs in plasma was more pronounced at 40°C and 42°C, which corresponded to the linear dependence of plasma DNase I activity on temperature. Further addition of DNase I also boosted the degradation of NETs at all temperatures, except for 35°C.

## 4 Discussion

Induction of NETs is beneficial in the early stages of systemic infection when they can prevent pathogen dissemination but becomes a dangerous burden when they are not efficiently cleared from the circulation (19, 60). In the present study, we show that both, NETs induction and NETs degradation are positively modulated by temperature up to 40°C, where it reaches its peak. At 35°C, however, the induction and degradation of NETs was attenuated, even after the addition of DNase I. This phenomenon holds for both infectious and sterile stimuli, highlighting the innate immune properties of PMNs in response to temperature. Moreover, we identified CD49d+ PMNs to exhibit high FPR1 expression, represent a minor population, but yet, make a major contribution to NETs formation.

Firstly, we found that DNA release in response to infectious (*E. coli, S. aureus*) and sterile (mitochondria) stimuli including PMA followed a similar bell-shaped pattern, with low DNA release at 35°C, progressive increase between 35-40°C and again minimal release at higher temperature. Interestingly, this was inversely related to the onset of NETs formation. These alterations in DNA release kinetics may thus contribute to the differences in PMN death observed at different temperatures, as delayed reaction to stimuli most likely results in smaller overall DNA release and vice versa.

We next modelled systemic infection and tissue damage in drawn peripheral blood and found that only CD16+ PMNs were responsible for NETs formation. In accordance with the *in vitro* studies, *ex vivo* NETs formation in response to all stimuli showed a similar pattern with maximal response at 40°C, and an attenuated response at 35°C and at 42°C. Dampened NETs formation at 42°C raised the hypothesis that extreme hyperthermia may instead of NETs formation drive neutrophils into apoptosis. Previous studies that have analyzed the effect of both short and long-term hyperthermia on PMNs viability found no effect of the increased temperature on spontaneous induction of apoptosis; we also observed no increase in the percentage of apoptotic cells in response to any of the stimuli, regardless of temperature (22, 61). Still, a limiting factor of *ex vivo* induced NETs formation is the lack of complexity achievable in this system. This approach does not reflect on the increased granulopoiesis in fever, nor takes into account the action of pyrogenic cytokines that mediate temperature increase in the brain. Future studies investigating the effect of temperature on NETs formation using animal models are warranted.

The fact that even *ex vivo* induced NETs formation is enhanced in fever and higher DNA release at 40°C supports the notion that systemic- independent mechanisms are at play. It is tempting to suggest that direct effect of temperature on NETosis kinetics may represent one such mechanism. Neubert et al. showed that the initial steps of NETosis are biochemically driven and dependent on the enzymatic activity of neutrophil elastase and MPO and both are accelerated at higher temperatures (62). They identified temperature-driven shortening of the initial, enzyme-dependent phase of NETosis with minimal effect on chromatin swelling or nuclear membrane rupture. Conversely, a lower temperature of 23.5°C significantly prolonged the whole process, which suggests that the speed of NETs formation is mainly dependent on the activity of enzymes involved. This would allow PMNs to respond to pathogen presence with accelerated NETs formation immediately after an increase in body temperature, without the need for any additional regulatory stimuli, as was observed in our model. It is important to note that next to enzymatic activity, increase in temperature also enhances the activation of ion channels that are implicated in neutrophil migration. It has been previously described that a Transient receptor potential cation channel, subfamily M, member 2 protein (TRPM2) that regulates calcium influx and controls neutrophil migration towards H_2_O_2_ is highly thermosensitive (63). Increased neutrophil chemotaxis during fever could then result in a quicker interaction of neutrophils with the pathogen and thus accelerate NETs induction.

The phenotype of neutrophils changes in response to pathogens during inflammation, as they age in circulation or certain pathologies. The β1 integrin (CD49d) that mediates neutrophil adhesion to endothelium and is in physiological conditions expressed on less than 10% of all neutrophils is present on one-third of neutrophils in patients with sepsis (64). Herein, we found minimal effect of temperature on CD49d expression in any of the control CD16+ PMNs. In mice, CD49d expression has been associated with the aged neutrophil phenotype and as shown by Zhang et al., aged neutrophils are more prone to NETs induction (36, 37, 65). We have also found that the majority of the NETs that were formed in response to all treatments originated from CD16+ CD49d+ PMNs even though only about 7-16% of total CD16+ PMNs expressed it. To investigate the possible reason why NETs formation capacity is higher in CD16+ PMNs expressing CD49d, we focused on receptors implicated in pathogen recognition.

PMA itself does not require a receptor, but both bacteria and mitochondria are recognized through FPR1 which detects formyl peptides, present either on their surface or released during septic shock (21, 39, 40). We have observed that CD16+ CD49d+ PMNs expressed about 5-times higher levels of FPR1 than CD49d- ones and this expression was maintained even in higher temperatures. The effect of temperature on the expression of FPR1 was to the best of our knowledge not previously described, but it is known that FPR1 is constitutively expressed on the surface of quiescent neutrophils and is rapidly upregulated in response to a variety of pro-inflammatory stimuli such as lipopolysaccharide (LPS) (66). Indeed, CD16+ CD49d- PMNs increased the expression of FPR1 only in response to *E. coli* and this change was prominent at all temperatures up to 42°C. *E. coli* contains LPS, which is like the prototypical FPR1 ligand fMLF (N-formyl-Met-Leu-Phe) a poor inducer of NETs formation by itself, but it is intriguing that these molecules can in combination induce extensive production of ROS and activate NADPH oxidase, an enzyme critical for NETs induction (67). Interestingly, *E. coli* together with PMA induced overall more NETs than *S. aureus* or mitochondria, which can be possibly attributed to the increased activation of CD16+ CD49d- PMNs, as the response of CD16+ CD49d+ PMNs was constant across all tested stimuli. Conversely, mitochondria dramatically increased FPR1 expression in CD16+ CD49d+ PMNs at both 40°C and 42°C, suggesting a distinct mechanism of neutrophil response to infectious (i.e. gram-negative bacteria) and sterile stimuli based on PMN phenotype. What is the implication of this observation needs to be further determined, possibly by blocking FPR1 with specific antagonists or antibodies. It can however be hypothesized that in case of sterile injury, targeting CD16+ CD49d+ PMNs with high expression of FPR1 may prove to be a viable strategy to reduce unnecessary NETs formation.

FPR1 is implicated in many other neutrophil functions and its activation has also been found important for the phagocytosis of *E. coli* and *S. aureus* (68, 69). Since cytoskeleton degradation is required for NETs formation but its integrity is critical for effective phagocytosis, it has been proposed that these two mechanisms are mutually exclusive (41, 70). It is interesting to note that the effect of temperature on neutrophil phagocytosis is not clear at all and the studies that have been done so far yield conflicting results. Several report a downregulation of phagocytosis in short-term fever range hyperthermia, both in response to gram-negative (*Pseudomonas aeruginosa, E. coli*) and gram-positive bacteria (*S. aureus*), some have shown its increase (22, 71, 72).

We recorded an increase in phagocytosis of both *E. coli* and *S. aureus*, between 37°C, 40°C and 42°C while no change was observed at 35°C, whilst mitochondria were barely phagocytosed. This was in contrast with the proposed hypothesis, however, the relationship between phagocytosis and NETs formation may not be so simple. Saffarzadeh and colleagues have proposed a non-canonical pathway where antibody- or complement-induced phagocytosis triggers rapid NETs formation independent of NADPH oxidase and a work by Ullah and colleagues showed that autophagy-enhanced phagocytosis of *Streptococcus pneumoniae* increased NETs formation (73, 74). Since phagocytosis happens within minutes and NETs formation requires >1 hour, it can also be argued that a phagocyting neutrophil can later undergo NETs formation, when the relevant pathways are activated. As with FPR1, CD11b is lowly expressed on resting PMNs and is upregulated after exposure to bacterial endotoxins like LPS (42). In our study, only PMNs treated with *E. coli* upregulated surface CD11b expression that was maximum at 35°C and with reduction with increasing temperature up to 42°C, where the difference was no longer significant. Upregulation of CD11b can thus explain the increased phagocytosis of *E. coli* in comparison to *S. aureus* and mitochondria, but not the pattern observed across different temperatures.

Pro-inflammatory cytokines released during systemic inflammation can also affect the activity of PMNs and it has been previously shown that high concentrations of IL-1β and TNF-α potentiate NETs formation (50, 51). Infectious stimuli induced significant release of IL-1β, TNF-α, IL-6 and IL-8, but changes in temperature between 35-40°C had no effect on their concentration and therefore cannot explain the differences in NETs formation. Keitelman and colleagues conversely reported that short-term incubation of neutrophils with *P. aeruginosa* at fever range hyperthermia results in a decrease in IL-1β, TNF-α and IL-8 (22). Neutrophils are not potent producers of cytokines and are not major contributors to the overall cytokine milieu, which may explain the discrepancies between studies. Interestingly, mitochondria induced a comparable release of said pro-inflammatory cytokines, but only at 37°C and the response at both, higher and lower temperatures was attenuated. The implication of this observation remains to be determined, but it suggests different regulation of immune response during sterile and pathogen-induced fever.

Excessive NET formation is proinflammatory in nature and NETs and their components have been described to activate immunity via several receptors including TLR4, TLR8 and TLR9 (55, 56, 75, 76). This can initiate a vicious circle in sepsis, where NETs activate neutrophils leading to the formation of more NETs. TLR9 activation is not dependent only on the concentration of DNA but also on its length and shorter DNA fragments have been found to augment TLR9-dependent signaling (57). Since we observed that at 35°C, the ratio of large to small NETs fragments was higher than at 40°C, we aimed to elucidate whether the NETs formed at 40°C have a higher capacity to activate NF-kB via TLR9 and thus also contribute to the increased NETs formation in febrile-range fever. We found that NETs formed at all temperatures exhibited equal capacity to activate TLR9-dependent NF-kB signaling. This suggests that accelerated NETs formation at 40°C was at least in the current experimental setting not potentiated by NETs themselves through TLR9. The role of TLRs in NET-driven inflammatory response during fever requires further investigation.

Next to pathogen entrapment, NETs also damage host tissue, therefore their degradation is as important as their formation (18–20). We observed a strong positive correlation between plasma nuclease activity and temperature, and NETs degradation was also enhanced at higher temperatures when compared to 37°C. Interestingly, the addition of DNase I further boosted NETs degradation at all temperatures except for 35°C, where the effect was not significant. We therefore propose a physiological mechanism where temperature modulates both the intensity of NETs formation and NETs clearance. Accelerated and more pronounced NETs formation during fever would reduce pathogen dissemination in early phase of the disease and initial rate of NET degradation may be a key determinant of clinical outcome and tissue damage. At the same time, hypothermia would result in a decreased pathogen entrapment due to its slower onset and NETs that would remain in the circulation would be cleared less efficiently even after therapeutic administration of nucleases. Our results would therefore be in agreement with clinical studies where 35°C was the upper limit of temperature not beneficial for the patient and febrile-range fever of 40°C resulted in their increased survival (7–10). Future studies must elucidate if the enhanced NETs turnover at higher temperatures reported herein can be manipulated for better outcome in animal models and appropriate patient cohorts.

Induction of fever is strongly associated with enhanced infection resolution but comes at a high metabolic cost and as such is often countered by drugs to lower body temperature (77). The use of antipyretics is however not always beneficial, as their administration was reported to be associated with increased mortality of intensive care unit patients (78–80). This phenomenon has also been observed in clinical trials investigating the effect of temperature regulation on the outcome of sepsis, with several being suspended due to the increased mortality of patients in hypothermia (7–10). Fever was in contrast associated with increased immune response and improved survival (3). In this *in vitro* study, we have found that an increase in temperature is positively associated with both NETs formation and degradation and propose that this phenomenon could contribute to the beneficial effect of fever on the survival of patients with sepsis. Whether these observations hold in a clinical setting needs validation.

## Data availability statement

The raw data supporting the conclusions of this article will be made available by the authors, without undue reservation.

## Ethics statement

The studies involving humans were approved by Ethics Committee of the University Hospital Bratislava (workplace Ruzinov under number EK 218/2020). The studies were conducted in accordance with the local legislation and institutional requirements. The participants provided their written informed consent to participate in this study.

## Author contributions

JJ: Data curation, Formal Analysis, Investigation, Methodology, Writing – original draft. EB: Formal Analysis, Investigation, Methodology, Writing – original draft. KK: Formal Analysis, Investigation, Methodology, Writing – original draft. BK: Formal Analysis, Investigation, Methodology, Writing – original draft. LH: Formal Analysis, Investigation, Methodology, Writing – original draft. PC: Conceptualization, Methodology, Resources, Supervision, Writing – review & editing. MB-E: Supervision, Writing – review & editing. MP: Conceptualization, Formal Analysis, Funding acquisition, Methodology, Project administration, Resources, Supervision, Writing – original draft.
