# Supplementary figures and images for: Neutrophil extracellular traps formation and clearance is enhanced in fever and attenuated in hypothermia
Source: Front Immunol. 2023 Oct 2;14:1257422. doi: 10.3389/fimmu.2023.1257422 (PMC10577177; doi:10.3389/fimmu.2023.1257422)

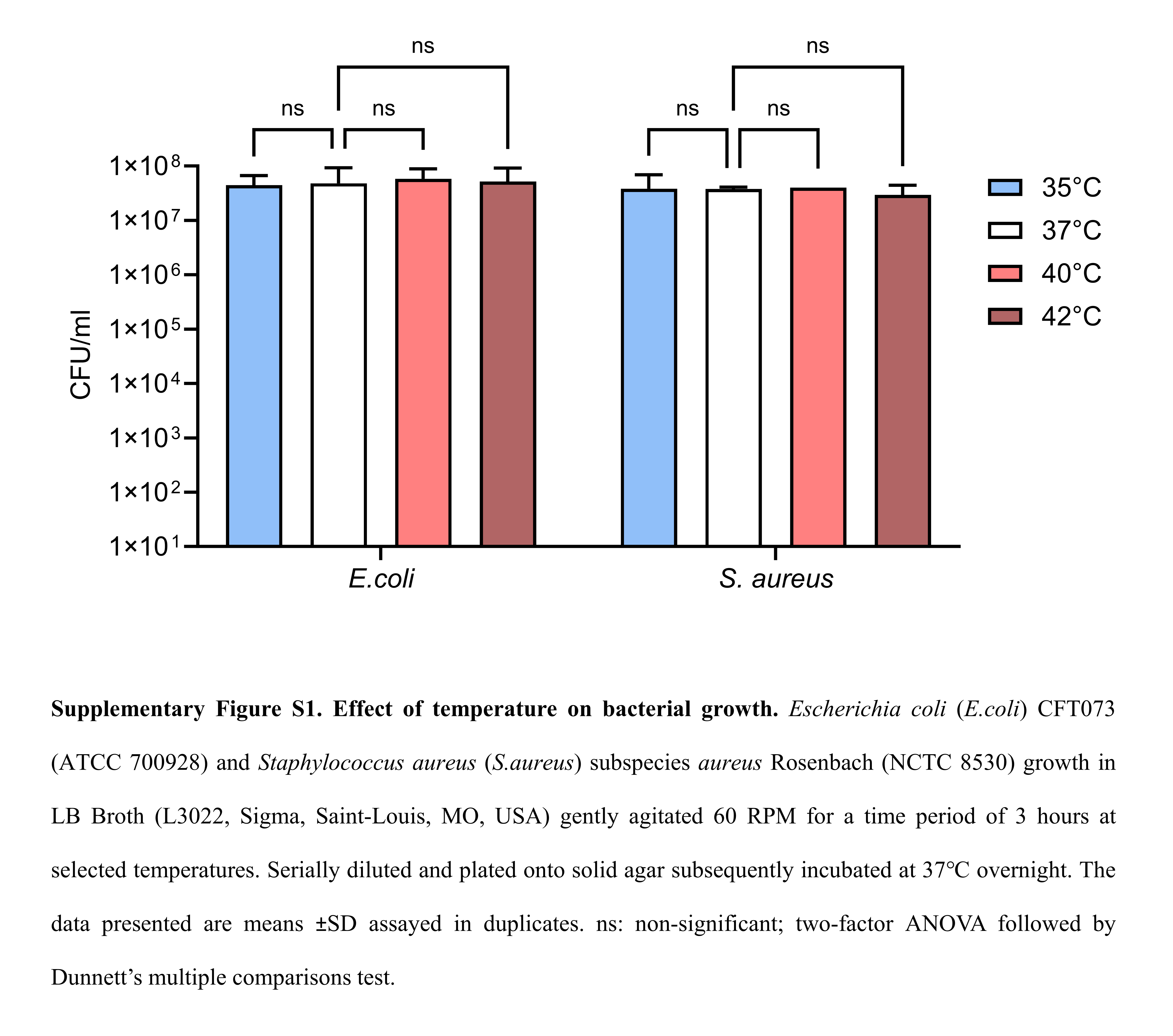

Supplement: Supplementary file 2 [file Image_1.tif]

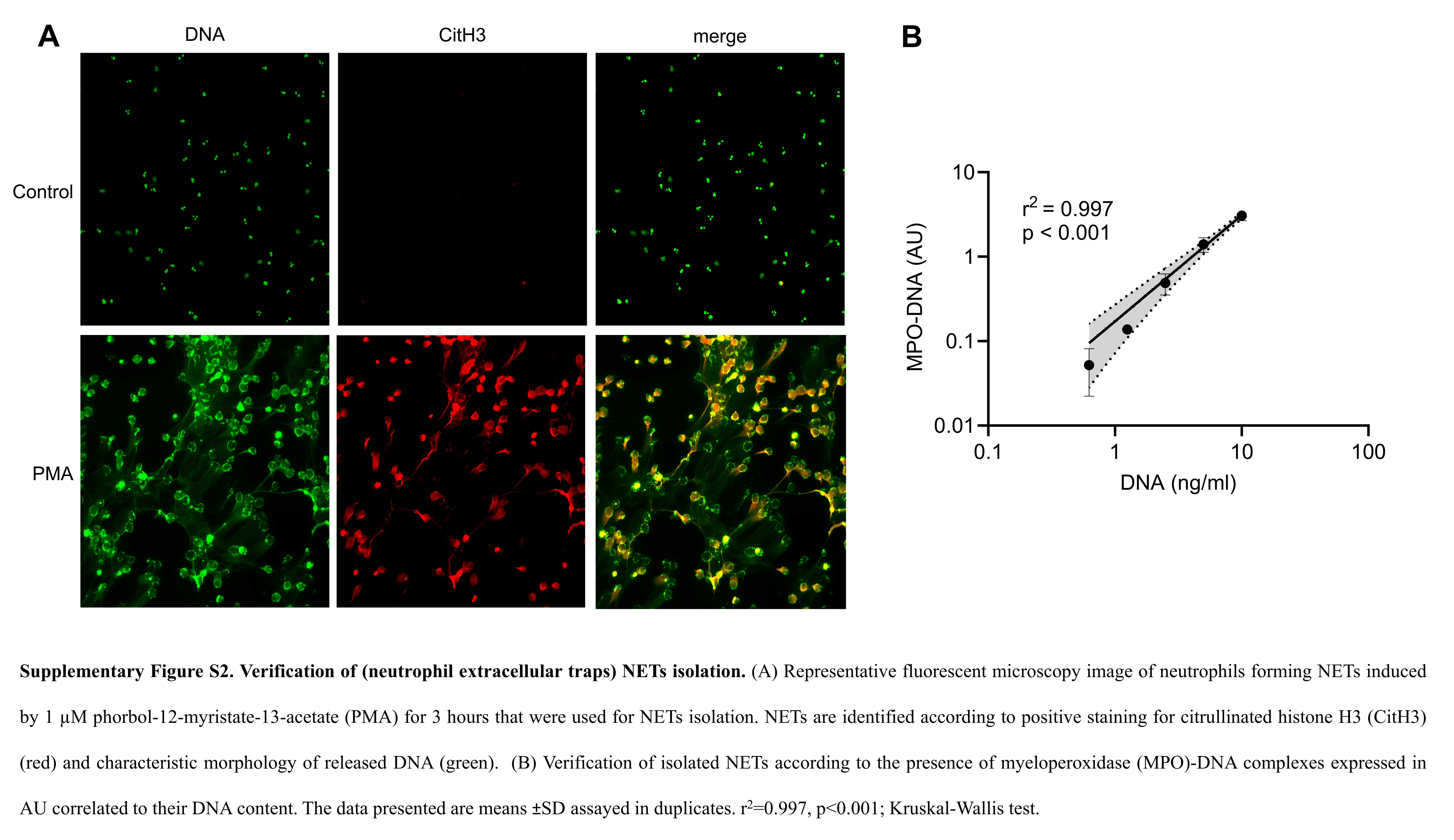

Supplement: Supplementary file 3 [file Image_2.tif]

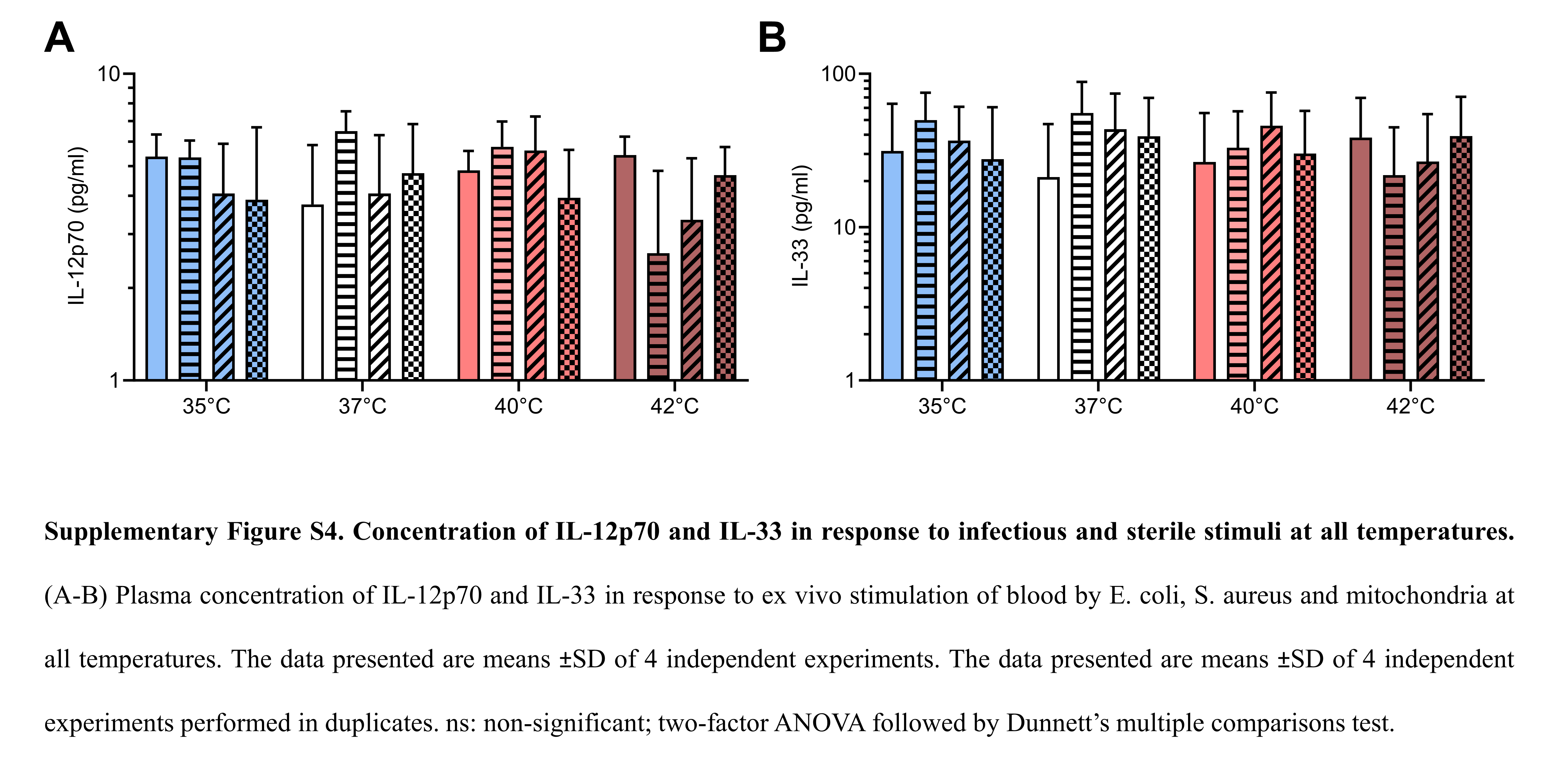

Supplement: Supplementary file 5 [file Image_4.tif]

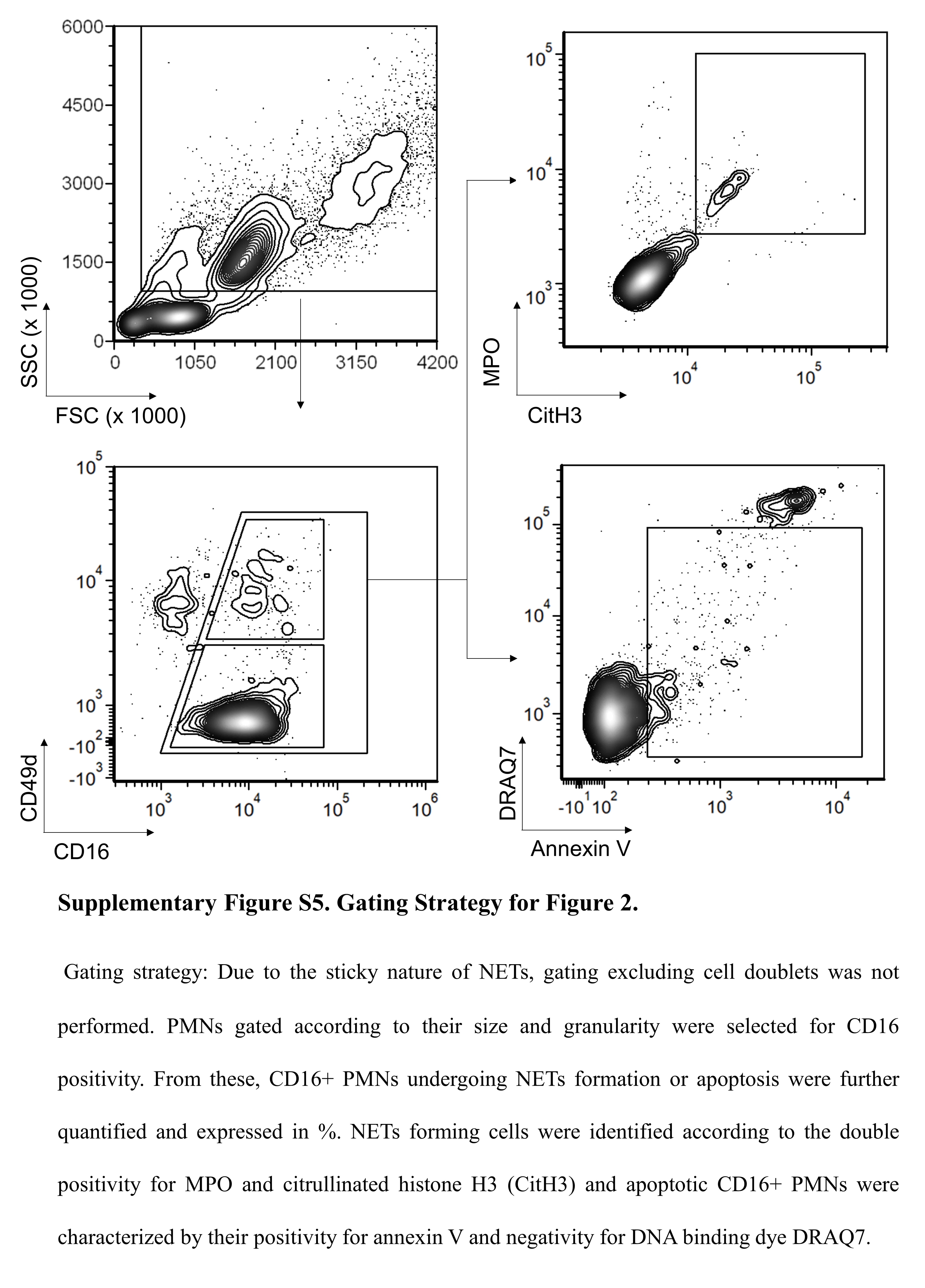

Supplement: Supplementary file 6 [file Image_5.tif]

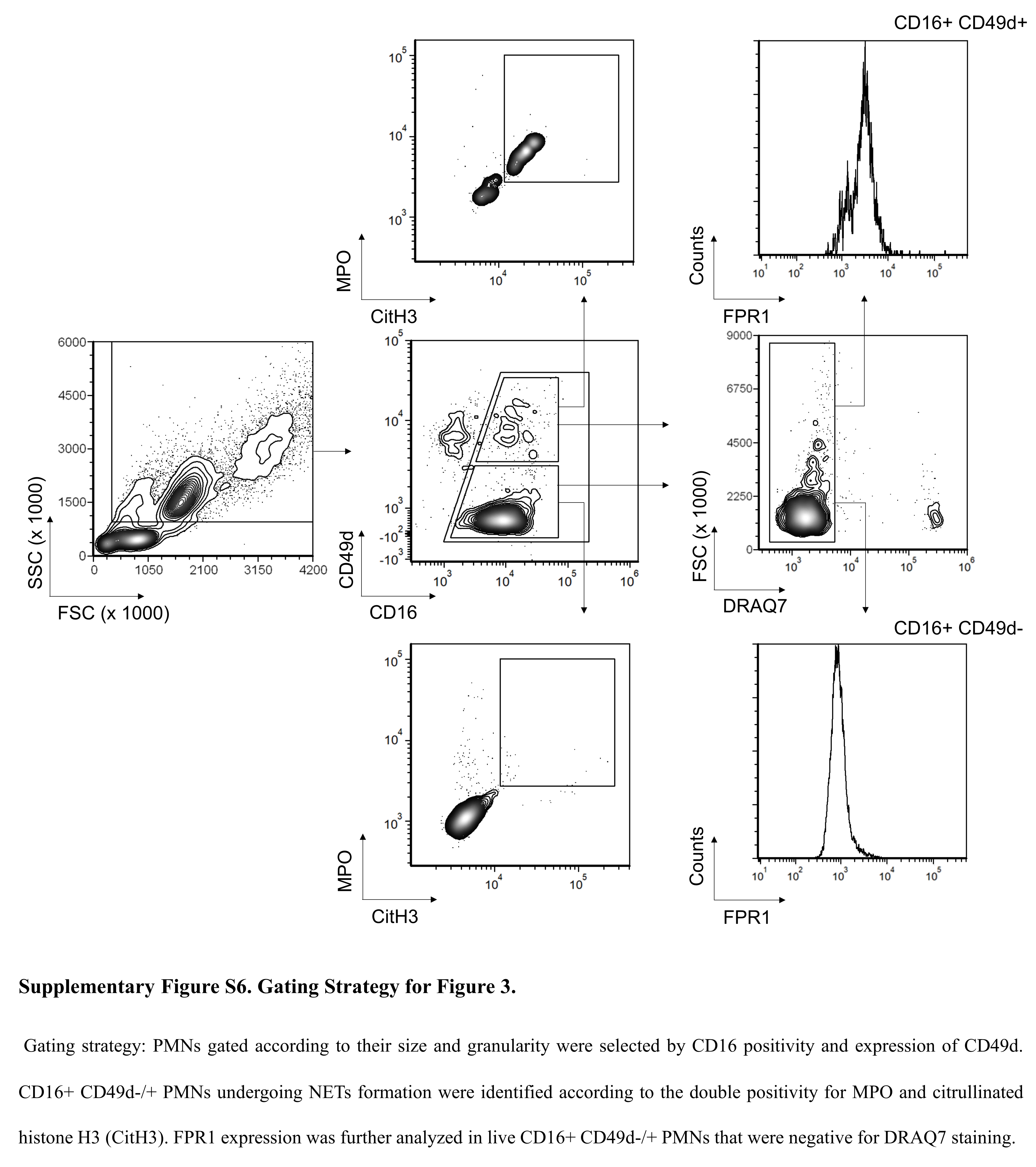

Supplement: Supplementary file 7 [file Image_6.tif]

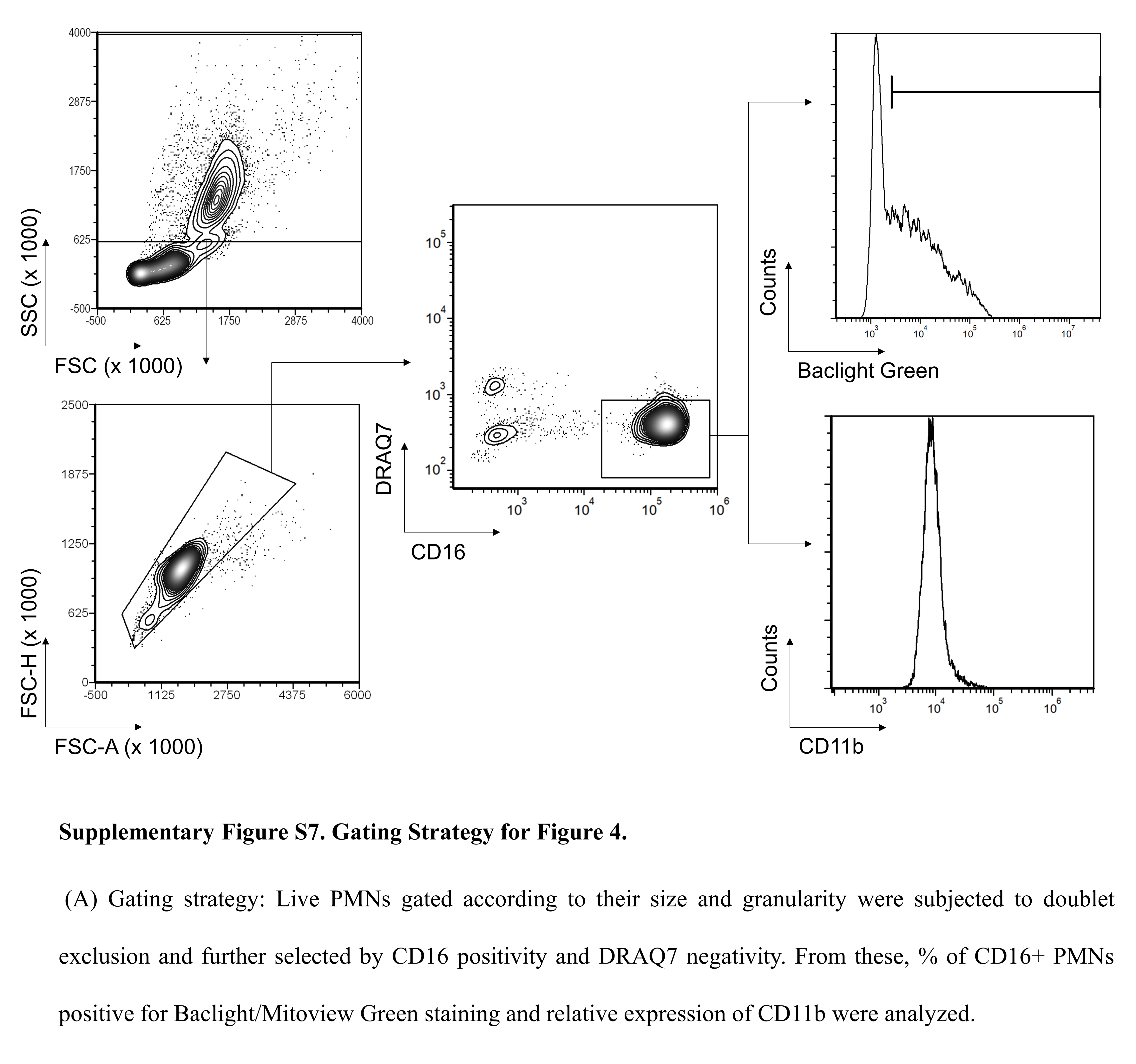

Supplement: Supplementary file 8 [file Image_7.tif]

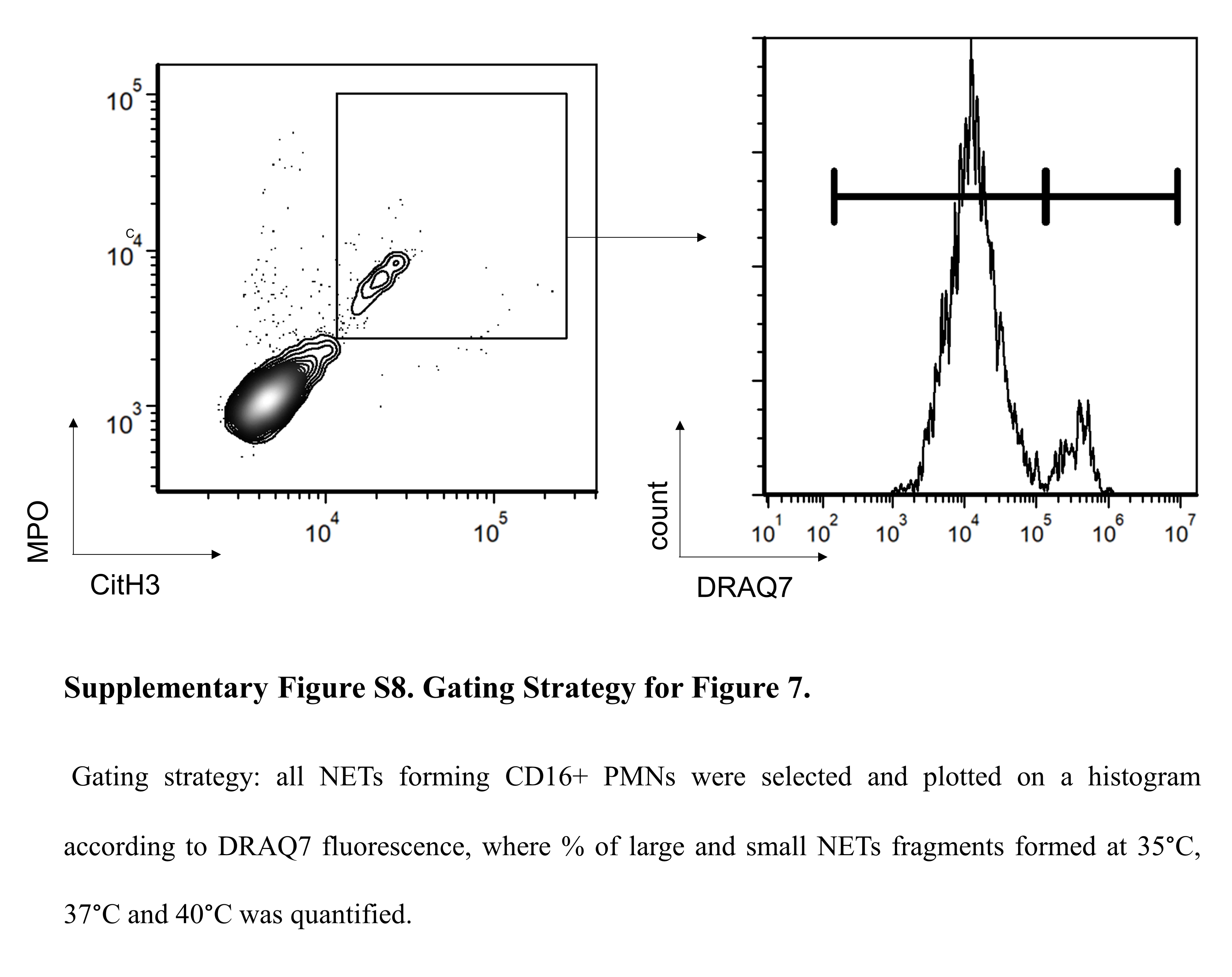

Supplement: Supplementary file 9 [file Image_8.tif]
